# Development and prospective validation of a risk score model in guiding individualized concurrent chemoradiotherapy in stage II nasopharyngeal carcinoma in intensity‐modulated radiotherapy era

**DOI:** 10.1002/cam4.4520

**Published:** 2021-12-24

**Authors:** Shan‐Shan Yang, Ya‐Jun Pang, Zhi‐Qiang Wang, Bao‐Yu Zhang, Zhi‐Qiao Liu, En‐Ni Chen, Pu‐Yun OuYang, Fang‐Yun Xie

**Affiliations:** ^1^ Department of Radiation Oncology Sun Yat‐sen University Cancer Center State Key Laboratory of Oncology in South China Collaborative Innovation Center for Cancer Medicine Guangdong Key Laboratory of Nasopharyngeal Carcinoma Diagnosis and Therapy Guangzhou China; ^2^ Cancer Center Affiliated Hospital of Guangdong Medical University Zhanjiang Guangdong Province China; ^3^ Department of Radiotherapy Affiliated Dongguan People's Hospital of Southern Medical University Dongguan People's Hospital Dongguan Guangdong China

**Keywords:** concurrent chemoradiotherapy, intensity‐modulated radiotherapy, nasopharyngeal carcinoma, tumor burden

## Abstract

**Purpose:**

We aimed to develop and prospectively validate a risk score model to guide individualized concurrent chemoradiotherapy (CCRT) for patients with stage II nasopharyngeal carcinoma (NPC) in intensity‐modulated radiotherapy (IMRT) era.

**Materials and Methods:**

In total, 1220 patients who received CCRT or IMRT alone were enrolled in this study, including a training cohort (*n* = 719), a validation cohort (*n* = 307), and a prospective test cohort (*n* = 194). Patients were stratified into different risk groups by a risk score model based on independent prognostic factors, which were developed in the training cohort. Survival rates were compared by the log‐rank test. The validation and prospective test cohorts were used for validation.

**Results:**

Total tumor volume, Epstein–Barr virus DNA, and lactate dehydrogenase were independent risk factors for failure‐free survival (FFS, all *p *< 0.05). A risk score model based on these three risk factors was developed to classify patients into low‐risk group (no risk factor, *n* = 337) and high‐risk group (one or more factors, *n* = 382) in the training cohort. In the high‐risk group, CCRT had better survival rates than IMRT alone (5‐year FFS: 82.6% vs. 74.0%, *p* = 0.028). However, there was no survival difference between CCRT and IMRT alone either in the whole training cohort (*p* = 0.15) or in the low‐risk group (*p* = 0.15). The results were verified in the validation and prospective test cohorts.

**Conclusion:**

A risk score model was developed and prospectively validated to precisely select high‐risk stage II NPC patients who can benefit from CCRT, and thus guided individualized treatment in IMRT era.

## Graphical abstract

Stage II nasopharyngeal carcinoma patients cannot benefit from concurrent chemoradiotherapy. A risk score model were developed and prospectively validated to precisely select high‐risk patients who can gain survival benefit from concurrent chemoradiotherapy, and thus guide individualized treatment in stage II nasopharyngeal carcinoma.

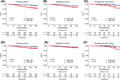

## 1 INTRODUCTION

Nasopharyngeal carcinoma is a unique head and neck cancer with unbalanced endemic distribution. Approximately 133,354 new patients were reported worldwide in 2020, with the high rates occurring in Southeastern Asia.^1^ Radiotherapy is the cornerstone of nasopharyngeal carcinoma treatment, due to its high sensitive to radiation. For stage I nasopharyngeal carcinoma, radiotherapy alone is the main curative treatment; however, whether stage II nasopharyngeal carcinoma could benefit from concurrent chemotherapy remains controversial. A prospective phase III study showed that concurrent chemoradiotherapy brought survival benefit for stage II nasopharyngeal carcinoma compared with two‐dimensional radiotherapy alone.^2^, ^3^ Since intensity‐modulated radiotherapy dramatically improved survival outcomes of nasopharyngeal carcinoma, omitting chemotherapy was considered for stage II patients in intensity‐modulated radiotherapy era. Several retrospective studies reported that concurrent chemoradiotherapy had comparable survival to intensity‐modulated radiotherapy alone for patients with stage II nasopharyngeal carcinoma,^4^, ^5^, ^6^ and a prospective study enrolling 84 patients also confirmed this conclusion.^7^ Intriguingly, a retrospective study based on National Cancer Database from the United States reported concurrent chemoradiotherapy improved survival of stage II nasopharyngeal carcinoma compared with intensity‐modulated radiotherapy alone.^8^ Similarly, a study demonstrated stage II nasopharyngeal carcinoma from nonendemic region could benefit from addition of concomitant chemotherapy in intensity‐modulated radiotherapy era.^9^ Given the inconsistent conclusions, finding a way to guide individualized treatment is needed.

Tumor volume was a significant prognostic factor in nasopharyngeal carcinoma. Lu et al.^10^ reported patients with gross tumor volume of nasopharynx higher than 20 ml had lower survival rates. Likewise, gross volume of lymph node had significant prognostic value in nasopharyngeal carcinoma,^11^, ^12^, ^13^, ^14^ and another study showed total tumor volume as an independent prognostic factor can improve prognostic validity of clinical stage.^15^ Pretreatment plasma Epstein–Barr virus (EBV) DNA has been widely used for prognosis and risk stratification. When tumor volume combined with EBV DNA, it can be better used for risk stratification in nasopharyngeal carcinoma.^10^, ^12^, ^14^ A recent study developed an integrated gross tumor value of cervical lymph node and EBV DNA model to predict survival and guide treatment for patients receiving induction chemotherapy.^14^ Chen et al. combined tumor volume and EBV DNA for prognostic stratification in patients with stage II nasopharyngeal carcinoma, however, patients who can gain survival benefit from concurrent chemoradiotherapy have not been identified in that study.^16^ Therefore, we aimed to develop and prospectively validate a risk score model to guide individualized concurrent chemoradiotherapy for stage II nasopharyngeal carcinoma patients in intensity‐modulated radiotherapy era.

## 2 MATERIALS AND METHODS

### 2.1 Patients and study design

**FIGURE 1:**
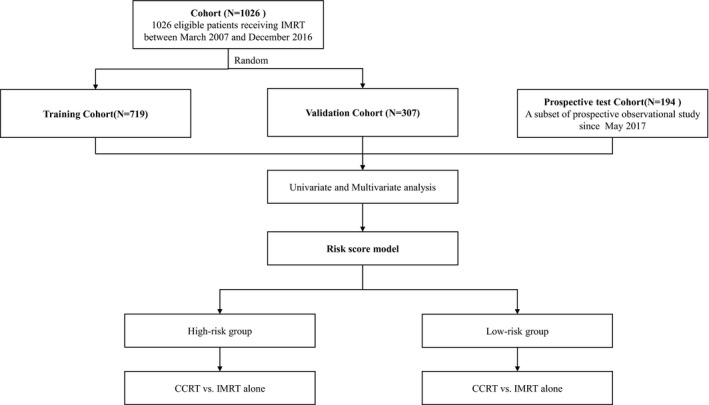
Flowchart of eligible patients. CCRT, concurrent chemoradiotherapy; IMRT, intensity‐modulated radiotherapy

A total of 1220 stage II nasopharyngeal carcinoma patients who received intensity‐modulated radiotherapy at Sun Yat‐sen University Cancer Center were included in this study. Among them, 1026 patients between March 2007 and December 2016 were randomly divided at ratio of 7:3 into a training cohort (*n* = 719) and a validation cohort (*n* = 307). And the prospective test cohort (*n* = 194) was a subset of our prospective observational study (Name: A Prospective Cohort Study of Nasopharyngeal Carcinoma to Establish Prognostic Models, to Discover Toxicity Associated Predictors and to Validate Randomized Trials in Clinical Practice. ClinicalTrials.gov Identifier: NCT03003182) since May 2017. The flow chart is shown in Figure 1. The clinical data were extracted from the nasopharyngeal carcinoma‐specific real‐world dataset based on a big‐data intelligence platform. The inclusion criteria were as follows: (1) newly diagnosed with stage II nasopharyngeal carcinoma including subgroups of T1N1M0, T2N0M0, and T2N1M0; (2) receipt of intensity‐modulated radiotherapy with or without concurrent chemotherapy; and (3) complete pretreatment head and neck magnetic resonance imaging (MRI) and EBV DNA. All patients were restaged according to eighth edition American Joint Committee on Cancer/Union for International Cancer Control staging system.

This study was approved by the institutional review board at Sun Yat‐sen University Cancer Center (No. B2020‐263) and informed consents were obtained.

### 2.2 Tumor volume measurement

All pretreatment head and neck MRI were obtained from picture archiving and communication system, and uploaded into ITK‐SNAP software (version 3.8.0; www.itksnap.org) to delineate regions of interest (ROIs). ROIs including the gross tumor volume of nasopharynx and lymph nodes were re‐outlined manually by a radiation oncologist (PYOY, 10 years of experience in contouring nasopharyngeal carcinoma) at each slice of axial contrast‐enhanced T1‐weighted, T1‐weighted, and T2‐weighted images (Figure S1) and checked by an expert radiation oncologist (FYX, over 30 years of experience in treating nasopharyngeal carcinoma). Then, the delineated gross tumor volumes were extracted by python software (https://github.com). The total tumor volume was equal to the gross tumor volume of nasopharynx plus gross tumor volume of lymph nodes. The protocol of MRI is deposited in Data S1.

### 2.3 Pretreatment plasma EBV DNA

Blood sample was gathered from enrolled patients for detection of plasma EBV DNA before treatment, and sample was measured by fluorescence polymerase chain reaction. The detailed method is shown in Data S1. And the cut‐off value of pretreatment plasma EBV DNA was defined as 2000 copy/ml based on previous study.^17^

### 2.4 Treatment and follow‐up

All eligible patients underwent radical intensity‐modulated radiotherapy. According to guideline, the prescribed doses of primary tumor and metastatic lymph nodes were 66–72 Gy and 64–70 Gy, respectively. Concurrent chemotherapy plan included weekly (30–40 mg/m^2^) or three‐weekly (80–100 mg/m^2^) cisplatin regimen. The detailed treatment is described in Data S1.

Follow‐up was conducted every 3–6 months during the first 2 years, and every 6 months to 1 year thereafter. During the follow‐up period, the routine examinations included nasopharyngoscopy, plasma EBV DNA, MRI, and computed tomography of chest and abdomen. And fluorine‐18‐fluorodeoxyglucose positron emission tomography/computed tomography and/or biopsy were conducted if necessary.

### 2.5 Statistical analysis

The primary endpoint was failure‐free survival (FFS), which was computed from treatment to failure (locoregional recurrence or distant metastasis). The secondary endpoint was locoregional relapse‐free survival (LRFS, defined as the time from treatment to locoregional recurrence).

The cut‐off values of continuous variables were determined by time‐dependent receiver operating characteristic (ROC) curve analysis. Survival rates were calculated through the Kaplan–Meier method and compared by the log‐rank test. Univariate and multivariate Cox regression analyses were performed to select risk factors. The risk score model was developed in the training cohort, and validated in the validation and prospective test cohorts. Statistical analysis was conducted with SPSS 26.0 and R software (version 4.0.1; http://www.r‐project.org/). And a two‐sided *p *< 0.05 was defined as statistically significant.

## 3 RESULTS

### 3.1 Patient characteristics

A total of 1220 eligible patients were enrolled, including 719 patients in the training cohort, 307 patients in the validation cohort, and 194 patients in prospective test cohort. As shown in Table 1, patients with EBV DNA ≥2000 copies/ml counted for 49.7%, 45.3%, and 26.7% in the training, validation, and prospective test cohorts. The cut‐off value of total tumor volume was 11 ml for FFS (3‐year area under the curve [AUC]: 0.602, Figure S2) in the training cohort. With median follow‐up time of 76, 77, and 30 months in the training, validation, and prospective test cohorts, 15.6% (112/719), 14.0% (43/307), and 6.2% (12/194) of patients suffered from treatment failure. The 3‐year FFS was 91.9%, 94.0%, and 88.7% in the training, validation, and prospective test cohorts, respectively.

**TABLE 1:** Baseline characteristics of high‐risk and low‐risk patients in the training, validation, and prospective test cohorts

	Training cohort		*p*	Validation cohort		*p*	Prospective test cohort		*p*
	Low‐risk group	High‐risk group		Low‐risk group	High‐risk group		Low‐risk group	High‐risk group	
	*N* = 337	*N* = 382		*N* = 146	*N* = 161		*N* = 119	*N* = 75	
	*N* (%)	*N* (%)		*N* (%)	*N* (%)		*N* (%)	*N* (%)	
Sex			0.676			0.053			0.862
Female	101 (30.0)	108 (28.3)		50 (34.2)	38 (23.6)		36 (30.3)	21 (28.0)	
Male	236 (70.0)	274 (71.7)		96 (65.8)	123 (76.4)		83 (69.7)	54 (72.0)	
Age			0.476			0.066			0.71
<45	174 (51.6)	186 (48.7)		77 (52.7)	67 (41.6)		51 (42.9)	35 (46.7)	
≥45	163 (48.4)	196 (51.3)		69 (47.3)	94 (58.4)		68 (57.1)	40 (53.3)	
T stage			0.961			0.523			0.073
T1	95 (28.2)	106 (27.7)		41 (28.1)	39 (24.2)		41 (34.5)	16 (21.3)	
T2	242 (71.8)	276 (72.3)		105 (71.9)	122 (75.8)		78 (65.5)	59 (78.7)	
*N* stage			<0.001			<0.001			0.006
N0	120 (35.6)	35 (9.2)		70 (47.9)	21 (13.0)		40 (33.6)	11 (14.7)	
N1	217 (64.4)	347 (90.8)		76 (52.1)	140 (87.0)		79 (66.4)	64 (85.3)	
Overall stage			<0.001			<0.001			<0.001
T1N1M0	95 (28.2)	106 (27.7)		41 (28.1)	39 (24.2)		41 (34.5)	16 (21.3)	
T2N0M0	120 (35.6)	35 (9.2)		70 (47.9)	21 (13.0)		40 (33.6)	11 (14.7)	
T2N1M0	122 (36.2)	241 (63.1)		35 (24.0)	101 (62.7)		38 (31.9)	48 (64.0)	
Pathology			0.531			0.522			1
WHO I	0 (0.0)	1 (0.3)							
WHO II	5 (1.5)	8 (2.1)		0 (0.0)	2 (1.2)		1 (0.8)	1 (1.3)	
WHO III	332 (98.5)	373 (97.6)		146 (100.0)	159 (98.8)		118 (99.2)	74 (98.7)	
EBV DNA (copy/ml)			<0.001			<0.001			<0.001
<2000	337 (100.0)	192 (50.3)		146 (100.0)	88 (54.7)		119 (100.0)	55 (73.3)	
≥2000	0 (0.0)	190 (49.7)		0 (0.0)	73 (45.3)		0 (0.0)	20 (26.7)	
Hemoglobin (g/L)			0.981			1			0.988
<120	28 (8.3)	33 (8.6)		11 (7.5)	12 (7.5)		5 (4.2)	4 (5.3)	
≥120	309 (91.7)	349 (91.4)		135 (92.5)	149 (92.5)		114 (95.8)	71 (94.7)	
LDH(U/L)			<0.001			0.004			0.003
<250	337 (100.0)	351 (91.9)		146 (100.0)	150 (93.2)		119 (100.0)	68 (90.7)	
≥250	0 (0.0)	31 (8.1)		0 (0.0)	11 (6.8)		0 (0.0)	7 (9.3)	
Total tumor volume(ml)			<0.001			<0.001			<0.001
<11	337 (100.0)	61 (16.0)		146 (100.0)	35 (21.7)		119 (100.0)	18 (24.0)	
≥11	0 (0.0)	321 (84.0)		0 (0.0)	126 (78.3)		0 (0.0)	57 (76.0)	
Treatment			<0.001			0.005			0.003
IMRT alone	159 (47.2)	110 (28.8)		61 (41.8)	42 (26.1)		62 (52.1)	22 (29.3)	
CCRT	178 (52.8)	272 (71.2)		85 (58.2)	119 (73.9)		57 (47.9)	53 (70.7)	

Abbreviations: CCRT, concurrent chemoradiotherapy; EBV, Epstein–Barr virus; IMRT, intensity‐modulated radiotherapy; LDH, lactate dehydrogenase; WHO, World Health Organization.

### 3.2 Risk score model and risk stratification

**FIGURE 2:**
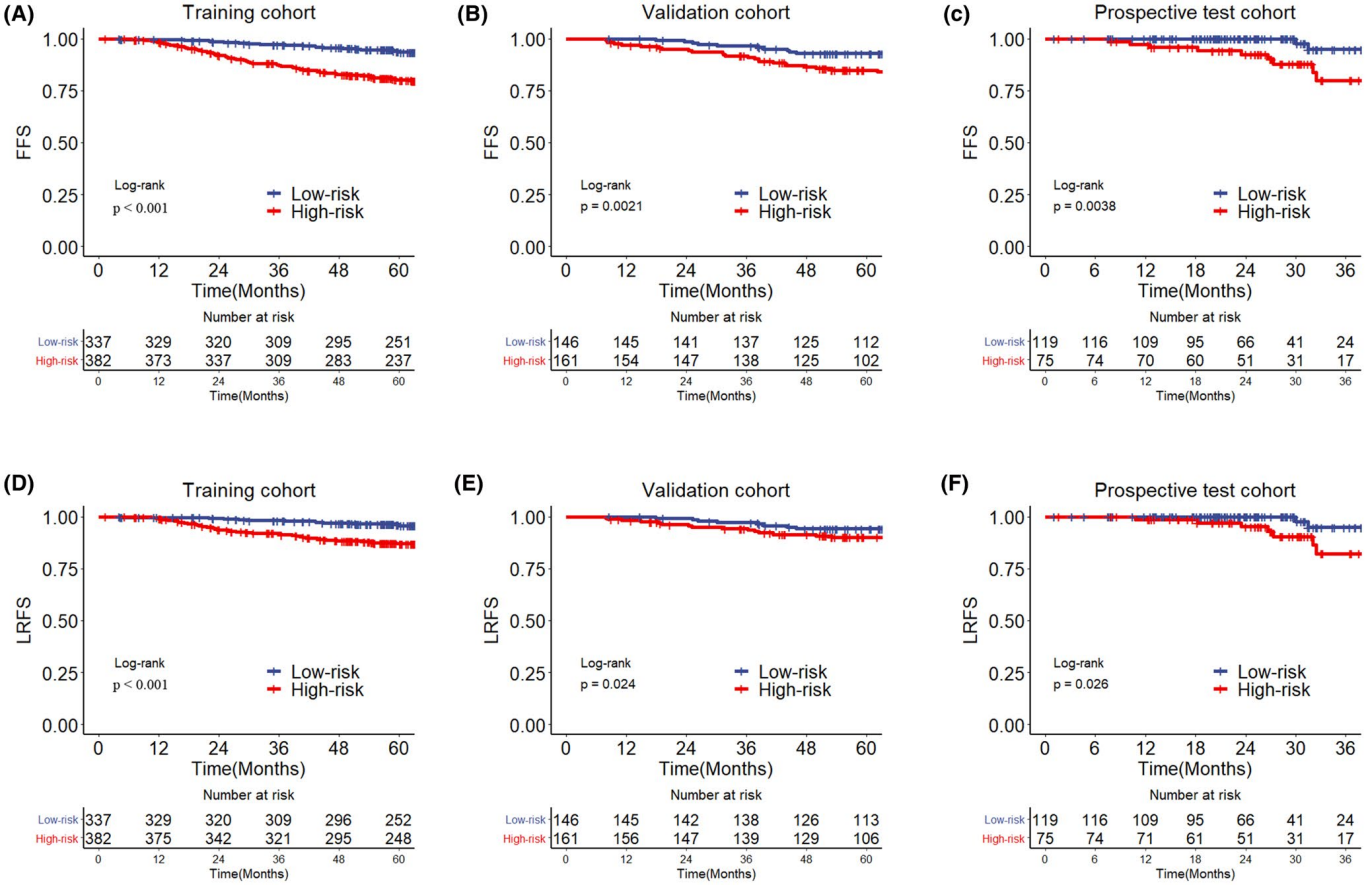
Survival curves of high‐risk group and low‐risk group in the training, validation, and prospective test cohorts. FFS, failure‐free survival; LRFS, locoregional relapse‐free survival

In the training cohort, univariate and multivariate Cox analyses showed EBV DNA, total tumor volume, and serum lactate dehydrogenase (LDH) were independent prognostic factors for FFS and LRFS (all *p *< 0.05, Table 2). In order to facilitate clinical application, one risk factor was scored one point. Thus, patients were scored from 0 to 3 points according to the number of risk factors. Finally, 336 (46.7%), 231 (32.1%), 142 (19.7%), and 10 (1.4%) patients had 0, 1, 2, and 3 points, respectively. Interestingly, survival curves showed patients with higher risk points had the lower survival rates (all *p *< 0.05, Figure S3). Subsequently, patients with risk score equal to 0 point were divided into low‐risk group, while patients with risk score higher than 0 point were stratified into high‐risk group. Hence, 382,161, and 75 patients were divided into high‐risk group, while 337,146, and 119 patients were split into low‐risk group in the training, validation, and prospective test cohorts, respectively. The baseline characteristics of two risk groups are presented in Table 1. And patients in the low‐risk group had higher survival rates than those in the high‐risk group in all cohorts (5‐year FFS in the training cohort: 93.5% vs. 80.1%, *p *< 0.001; 5‐year FFS in the validation cohort: 92.9% vs. 84.9%, *p* = 0.002; and 3‐year FFS in the prospective cohort: 94.9% vs. 79.9%, *p* = 0.0038; Figure 2).

**TABLE 2:** Univariate analysis and multivariate analysis for FFS and LRFS in the training cohort (*N* = 719)

	FFS				LRFS			
	Univariate analysis		Multivariate analysis		Univariate analysis		Multivariate analysis	
	HR (95% CI)	*p*	HR (95% CI)	*p*	HR (95% CI)	*p*	HR (95% CI)	*p*
Sex (Male vs. female)	1.19 (0.78–1.80)	0.425			1.13 (0.68–1.88)	0.629		
Age (≥45 vs. <45)	1.05 (0.72–1.52)	0.811			0.86 (0.55–1.36)	0.530		
T stage (T2 vs. T1)	0.97 (0.45–1.46)	0.885			0.87 (0.54–1.42)	0.585		
*N* stage (N1 vs. N0)	1.11 (0.70–1.77)	0.663			1.15 (0.64–2.06)	0.636		
Pathology	0.59 (0.23–1.49)	0.264			0.74 (0.20–2.80)	0.657		
EBV DNA (≥2000 vs. <2000)	2.32 (1.59–3.37)	<0.001	1.80 (1.20–2.69)	0.004	2.29 (1.45–3.62)	<0.001	1.69 (1.04–2.74)	0.035
Hemoglobin (≥120 vs. <120)	1.42 (0.69–2.92)	0.345			1.60 (0.64–4.01)	0.312		
LDH (≥250 vs. <250)	4.72 (2.73–8.15)	<0.001	4.42 (2.55–7.65)	<0.001	3.37 (1.61–7.04)	0.001	3.24 (1.55–6.79)	0.002
Total tumor volume (≥11 vs. <11)	2.09 (1.43–3.06)	<0.001	1.72 (1.14–2.58)	0.009	2.52 (1.56–4.05)	<0.001	2.14 (1.29–3.55)	0.003
Treatment (CCRT vs. IMRT alone)	0.76 (0.52–1.10)	0.149			0.68 (0.43–1.08)	0.100		

Abbreviations: CCRT, concurrent chemoradiotherapy; CI, confidence interval; EBV, Epstein–Barr virus; FFS, failure‐free survival; HR, hazard ratio; IMRT, intensity‐modulated radiotherapy; LDH, lactate dehydrogenase; LRFS, locoregional relapse‐free survival.

### 3.3 Guiding individualized concurrent chemotherapy

**FIGURE 3:**
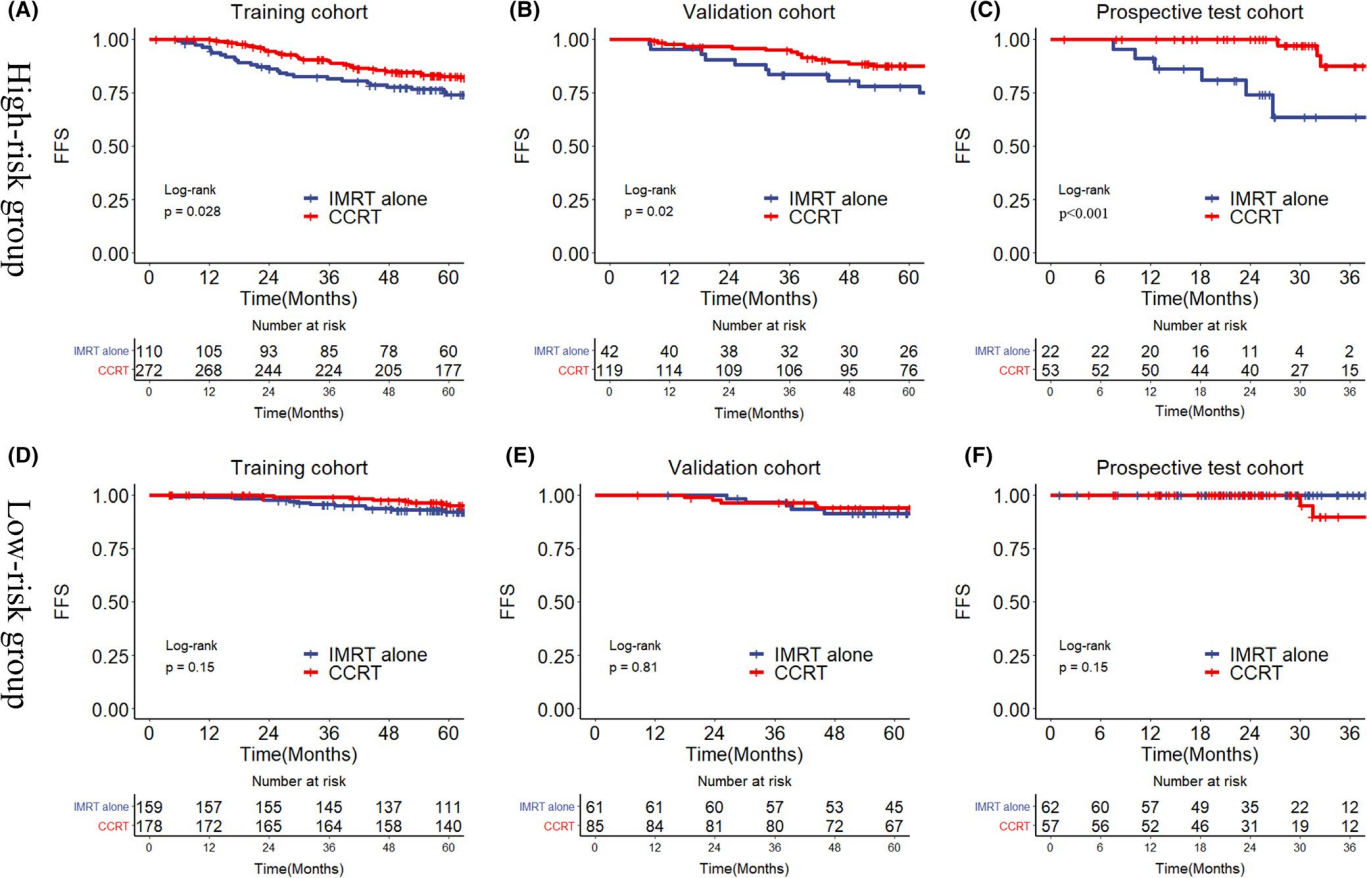
Survival curves of CCRT and IMRT alone for FFS in high‐risk (A–C) and low‐risk groups (D–F) of training, validation, and prospective test cohorts. CCRT, concurrent chemoradiotherapy; FFS, failure‐free survival; IMRT, intensity‐modulated radiotherapy

For patients in the whole training, validation, and prospective test cohorts, concurrent chemoradiotherapy had comparable survival rates to intensity‐modulated radiotherapy alone (5‐year FFS in the training cohort: 87.5% vs. 84.8%, *p* = 0.15; 5‐year FFS in the validation cohort: 90.1% vs. 85.9%, *p* = 0.19; and 3‐year FFS in the prospective cohort: 88.6% vs. 90.3%, *p* = 0.35; Figure S4). However, in the high‐risk group of cohorts, patients who received concurrent chemoradiotherapy had better survival rate than patients who received intensity‐modulated radiotherapy alone (5‐year FFS in the training cohort: 82.6% vs. 74.0%, *p* = 0.028; 5‐year FFS in the validation cohort: 87.5% vs. 77.9%, *p* = 0.02; and 3‐year FFS in the prospective cohort: 97.1% vs. 63.4%, *p *< 0.001; Figure 3A–C). Multivariate cox regression analysis also indicated concurrent chemoradiotherapy was a favorable prognostic factor in the high‐risk group of training, validation, and prospective test cohorts (*p* = 0.03, *p* = 0.023, and *p* = 0.005; Table 3). On the contrary, there was no significant survival difference between concurrent chemoradiotherapy and intensity‐modulated radiotherapy alone in the low‐risk group of all cohorts (all *p *≥ 0.17, Figure 3D–F).

**TABLE 3:** Survival analysis of CCRT and IMRT alone in the high‐risk group of training, validation, and prospective test cohorts

	FFS		LRFS	
	HR (95% CI)	*p*	HR (95% CI)	*p*
Training cohort				
CCRT versus IMRT	0.61 (0.39–0.95)	0.030	0.54 (0.31–0.92)	0.023
Validation cohort				
CCRT versus IMRT	0.44 (0.21–0.89)	0.023	0.38 (0.16–0.92)	0.033
Prospective test cohort				
CCRT versus IMRT	0.11 (0.03–0.46)	0.003	0.15 (0.03–0.69)	0.015

Abbreviations: CCRT, concurrent chemoradiotherapy; CI, confidence interval; FFS, failure‐free survival; HR, hazard ratio; IMRT, intensity‐modulated radiotherapy; LRFS, locoregional relapse‐free survival.

Same conclusion was also achieved in LRFS. Only in the high‐risk group of all cohorts, concurrent chemoradiotherapy had survival benefits compared with intensity‐modulated radiotherapy alone (all *p *≤ 0.027; Figure S5). Also, multivariate analysis showed concurrent chemoradiotherapy was an independent prognostic factor for LRFS (*p* = 0.023, *p* = 0.033, and *p* = 0.015; Table 3) in the high‐risk group of training, validation, and prospective test cohorts.

### 3.4 Subgroup analysis

Subgroup analysis was conducted in all stage II nasopharyngeal carcinoma patients. In this study, 338 (27.7%), 297 (24.3%), and 585 (48.0%) patients were diagnosed with T1N1M0, T2N0M0, and T2N0 (Table S1). Among the whole cohort, 65.5% of distant metastases happened in T2N1M0 subgroup. Figure S6 shows that T2N1M0 subgroup had poor distant metastasis‐free survival (DMFS) than T1N1M0 and T2N0M0 subgroups (*p* = 0.041, *p* = 0.026). Subsequently, three subgroups were also stratified into high‐risk and low‐risk groups based on established risk score model. As presented in Figure S7, patients receiving concurrent chemoradiotherapy had similar FFS compared with those undergoing intensity‐modulated radiotherapy alone in the low‐risk group, regardless of clinical stage subgroup (all *p *≥ 0.091). However, concurrent chemoradiotherapy had better FFS than intensity‐modulated radiotherapy alone in the high‐risk T2N1M0 subgroup (5‐year FFS: 86.5% vs. 76.4%, *p* = 0.012), but not in the high‐risk T1N1M0 or high‐risk T2N0M0 subgroup. Multivariate analysis of high‐risk T2N1M0 subgroup demonstrated concurrent chemoradiotherapy was an independent prognostic factor for survival (HR: 0.51, 95% CI: 0.30–0.85, *p* = 0.0095).

## 4 DISCUSSION

In this large cohort study, a risk score model was developed and prospectively validated based on risk factors including total tumor volume, EBV DNA, and LDH, which were independent prognostic factors for FFS in stage II nasopharyngeal carcinoma patients. Correspondingly, high‐risk and low‐risk patients were stratified by this risk score model. And high‐risk stage II nasopharyngeal carcinoma patients who can benefit from concurrent chemotherapy were identified in the intensity‐modulated radiotherapy era.

In conventional radiotherapy era, a phase III randomized clinical trial proved that concurrent chemoradiotherapy had better survival than radiotherapy alone in stage II nasopharyngeal carcinoma. And the 10‐year outcome of this phase III trial further confirmed the survival benefit of concurrent chemoradiotherapy without adding late toxicities.^2^, ^3^ But in intensity‐modulated radiotherapy era, the benefit of concurrent chemotherapy was in debate due to lack of phase III trial. Herein, concurrent chemoradiotherapy did not have better survival outcomes than intensity‐modulated radiotherapy alone in the whole stage II nasopharyngeal carcinoma cohort, which was consistent with previous studies.^4^, ^7^, ^18^ However, a retrospective study of the United States showed addition of concurrent chemotherapy improved survival in stage II nasopharyngeal carcinoma. But significantly, patients undergoing radiotherapy alone tended to be older than those receiving concurrent chemoradiotherapy in that study, and elder patients were more likely to have poor survival. Thus, the potential covariates effected the results of this retrospective study of national cancer database in the United States.^8^ Equally, Luo et al^9^ found concurrent chemoradiotherapy had better 3‐year survival rate than radiotherapy alone in T2N1M0 nasopharyngeal carcinoma. It should be noted all patients were from nonendemic region, with WHO II the most common histological type, which may have significant differences from WHO III dominated histological type in our study. Therefore, whether above nonendemic retrospective studies can be extrapolated to patients in endemic region needed further investigation. What is more, the possible reason for inconsistence in above study may be that therapeutic decision was just based on TNM staging system. Considering heterogeneity of stage II nasopharyngeal carcinoma, individualized chemotherapy was necessary.

Total tumor volume, which carried tumor load information, was widely used for prognosis and risk stratification in nasopharyngeal carcinoma.^15^, ^16^ A large tumor volume was prone to tumor hypoxia, leading to chemotherapy and radiotherapy resistance, and had a high possibility of micro‐metastasis.^19^ Thus, it was reasonable that total tumor volume bigger than 11 ml was a poor prognostic factor in our study. Furthermore, the tumor volume was re‐outlined manually on pretreatment MRI and then extracted through software, rather than directly obtained from intensity‐modulated radiotherapy planning system. So, our tumor volume measurement method was more precise, with median total tumor volume smaller than that of previous study.^16^ EBV DNA has been widely and extensively used in diagnosis, risk stratification, and guiding individualized treatment, especially in nasopharyngeal carcinoma endemic region.^20^ Combining time‐dependent ROC analysis and previous study,^17^ the cut‐off value of pretreatment plasma EBV DNA was defined as 2000 copies/ml in our study. Patients with EBV DNA ≥2000 copies/ml did have poor survival rates in stage II nasopharyngeal carcinoma. Aside from total tumor volume and EBV DNA, elevated LDH was also related to higher tumor burden and more tumor angiogenesis, and predicted poor survival in various tumor, including nasopharyngeal carcinoma.^21^ In this study, LDH was further proved to be an independent prognostic factor. Consequently, taking these three risk factors into consideration, a risk score model was developed for risk stratification. Then, the value of concurrent chemotherapy was investigated in different risk groups. Interestingly, our study found concurrent chemoradiotherapy improved survival outcomes compared with intensity‐modulated radiotherapy alone in the high‐risk groups of all cohorts. As presented in our study, 5‐year FFS of high‐risk stage II nasopharyngeal carcinoma was similar to those of locoregionally advanced nasopharyngeal carcinoma.^22^ Considering the confirmed benefit of concurrent chemoradiotherapy in locoregionally advanced nasopharyngeal carcinoma, it was highly acceptable that patients receiving concurrent chemoradiotherapy had better survival outcomes than those undergoing radiotherapy alone in the high‐risk group. And our findings were verified in the validation cohort and prospective test cohort.

Similar to previous studies,^7^, ^23^ T2N1M0 subgroup had poor DMFS than other subgroups, and concurrent chemotherapy added no benefit for the whole T2N1M0 subgroup. After risk stratification, only high‐risk patients in the T2N1M0 subgroup, but not T1N1M0 or T2N0M0 subgroup, can benefit from addition of concurrent chemotherapy. Notably, since the sample size of high‐risk T1N1M0 and T2N1M0 subgroups was relatively small, T2N1M0 subgroup may not be the only group that can benefit from concurrent chemoradiotherapy in the stage II nasopharyngeal carcinoma. Thus, further investigation of these three subgroups was needed.

There are several advantages in this study. First of all, taking total tumor volume, EBV DNA, and LDH into account, a risk score model was developed and prospectively validated for selecting high‐risk patients who can benefit from concurrent chemoradiotherapy. Second, tumor volume measurement method was more precise than traditional methods. However, this study was from one center. And our multicenter, prospective phase III randomized clinical trial (NCT02610010) which aimed to compare concurrent chemoradiotherapy with intensity‐modulated radiotherapy alone in stage II nasopharyngeal carcinoma was ongoing.

In conclusion, stage II nasopharyngeal carcinoma patients cannot benefit from concurrent chemoradiotherapy. And a risk score model based on tumor burden was developed and prospectively validated to precisely select high‐risk patients who can gain survival benefit from concurrent chemoradiotherapy, and thus guided individualized treatment in stage II nasopharyngeal carcinoma.

## CONFLICT OF INTEREST

The authors have no conflict of interest.

## AUTHOR CONTRIBUTION

Concept and design: Yang, Ouyang, and Xie. Acquisition, analysis, or interpretation of data: All authors. Drafting of the manuscript: Yang, Pang, and Wang. Critical revision of the manuscript for important intellectual content: Ouyang, Xie, and Yang. Statistical analysis: All authors. Drafting the article: All authors.

## ETHICAL APPROVAL STATEMENT

This study was approved by the institutional review board at Sun Yat‐sen University Cancer Center (No. B2020‐263).

## Supporting information

Supplementary Material

## Data Availability

The data that support the findings of our study are available from the corresponding author upon reasonable request.
