# Supplementary material for: Development and prospective validation of a risk score model in guiding individualized concurrent chemoradiotherapy in stage II nasopharyngeal carcinoma in intensity‐modulated radiotherapy era
Source: Cancer Med. 2021 Dec 24;11(4):1109–18. doi: 10.1002/cam4.4520 (PMC8855903; doi:10.1002/cam4.4520)
Supplement: Supplementary file 1 — Supplementary Material [file CAM4-11-1109-s001.docx]

**Supplementary Method**

**MRI protocol**

All patients underwent head and neck MR imaging with a 1.5- or 3.0-T system (Signa CV/i, GE HealthCare, Chalfont St Giles, United Kingdom). The area from the suprasellar cistern to the inferior margin of the sternal end of the clavicle was scanned. T1-weighted fast spin-echo images in the axial, coronal, and sagittal planes (repetition time: 500–600 ms, echo time: 10–20 ms, and field of view: 22 cm); and T2-weighted fast spin-echo MR images in the axial plane (repetition time: 4000–6000 ms, echo time:95–110 ms, and field of view: 22 cm) were obtained before injection of contrast material. Spin-echo T1-weighted axial and sagittal sequences, and spin-echo T1-weighted fat-suppressed coronal sequences were performed after intravenous Gd-DTPA (Magnevist; Bayer Schering Pharma AG, Germany) injection at a dose of 0.1mmol/kg.

**Pretreatment plasma EBV DNA**

Before treatment, peripheral venous blood (3 ml) was gathered from eligible patients, saved in an EDTA tube and centrifuged at 3000 × *g* for 5min. Total plasma DNA was extracted using a QIAamp DNA Blood Mini Kit (Qiagen, Hilden, Germany). Fluorescence polymerase chain reaction (PCR) was performed using an EBV PCR quantitative diagnostic kit (Da-An Genetic Diagnostic Center, Guangzhou, China) targeting the BamHI-W region of the EBV genome. Data were analyzed using Applied Biosystems 7300 SDS software (Beijing, China). The undetectable EBV DNA was 0 copy/mL.

**Treatment and follow-up**

All patients received only intensity-modulated radiotherapy with or without concurrent chemotherapy. Delineation of target volumes was consistent with the International Commission on Radiation Units and Measurements Reports 50 and 62. The prescribed doses were 66–72 Gy/28–33 fractions to gross tumor and 64–70 Gy/28–33 fractions to lymph node, 60–63 Gy/28–33 fractions to the high-risk clinical target volume (CTV1), and 54–56 Gy/28–33 fractions to the low-risk clinical target volume (CTV2), respectively. Concurrent chemotherapy, weekly cisplatin (30–40 mg/m2), or 3-weekly cisplatin (80–100 mg/m2) regimen was administrated.

**S1 Fig.** Gross tumor volume and lymph node outlined on each slice of axial contrast-enhanced T1-weighted(a,d), T2-weighted(b,e), and T1-weighted images(c,f)


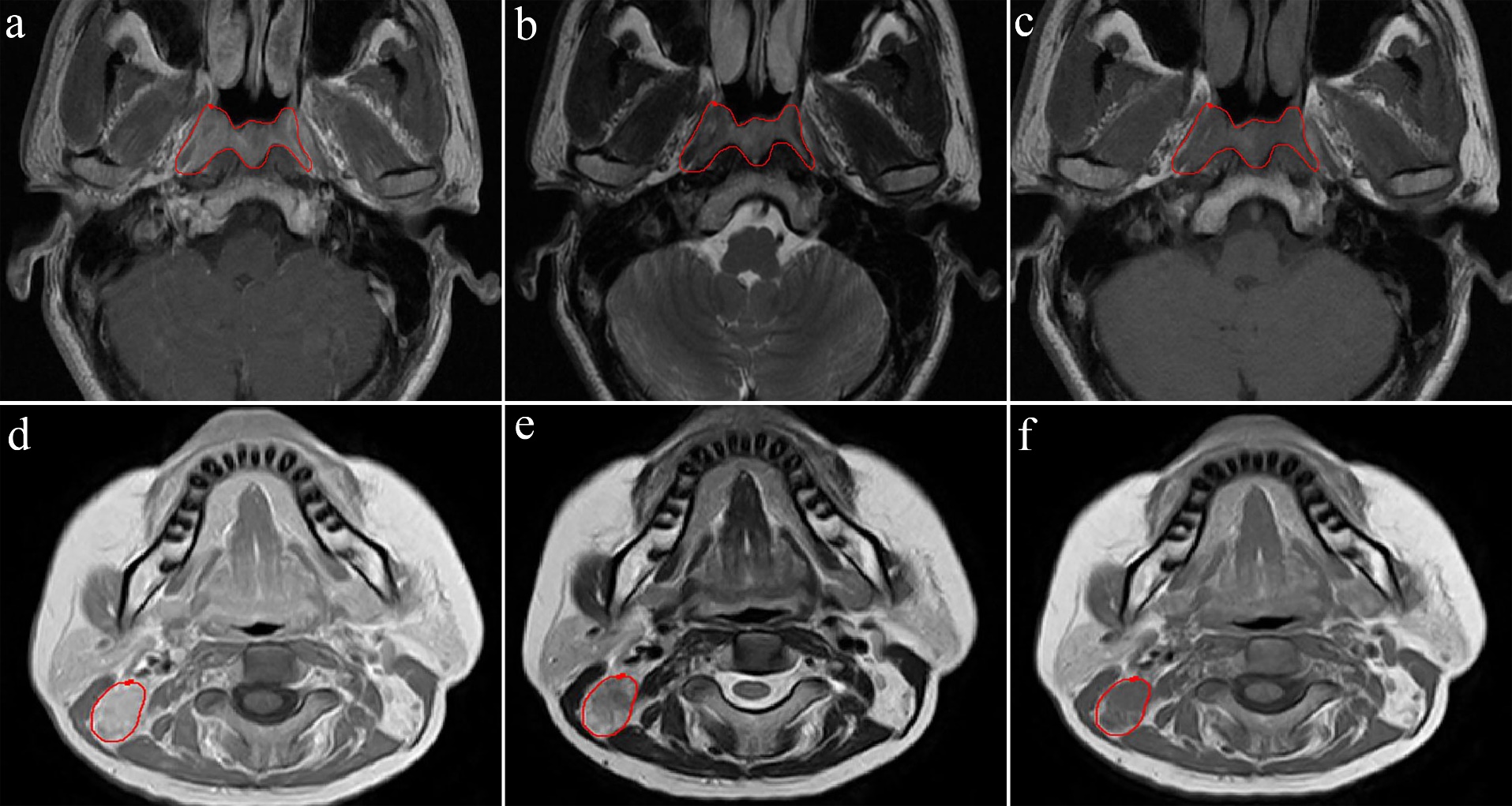


**S2 Fig.** Time-dependent receiver operating characteristic (ROC) curve of total tumor volume


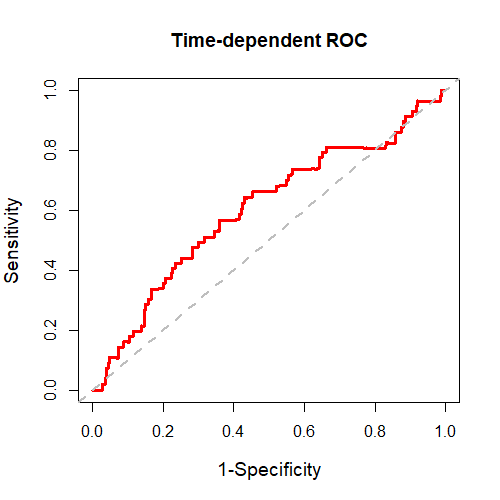


**S3 Fig.** Survival curves of risk scores for FFS(a) and LRFS(b) in the training cohort

Abbreviations: FFS, failure-free survival; LRFS, locoregional relapse-free survival.


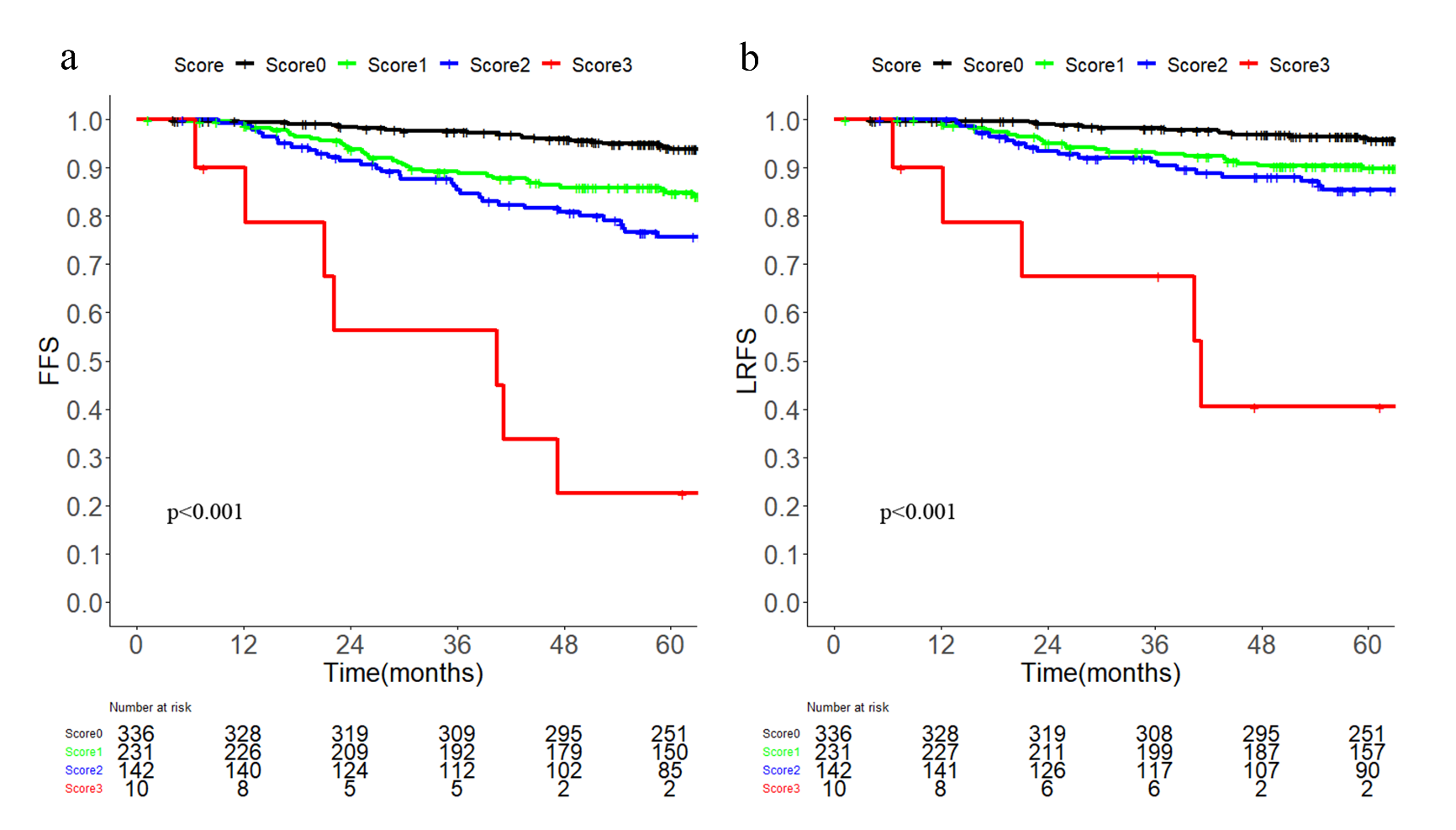


**S4 Fig.** Kaplan–Meier FFS(a-c), LRFS(d-f) curves of CCRT and IMRT alone in the whole training(a,d), validation(b,e), and prospective test cohort(c,f)

Abbreviations: CCRT, concurrent chemoradiotherapy; FFS, failure-free survival; IMRT, intensity-modulated radiotherapy; LRFS, locoregional relapse-free survival


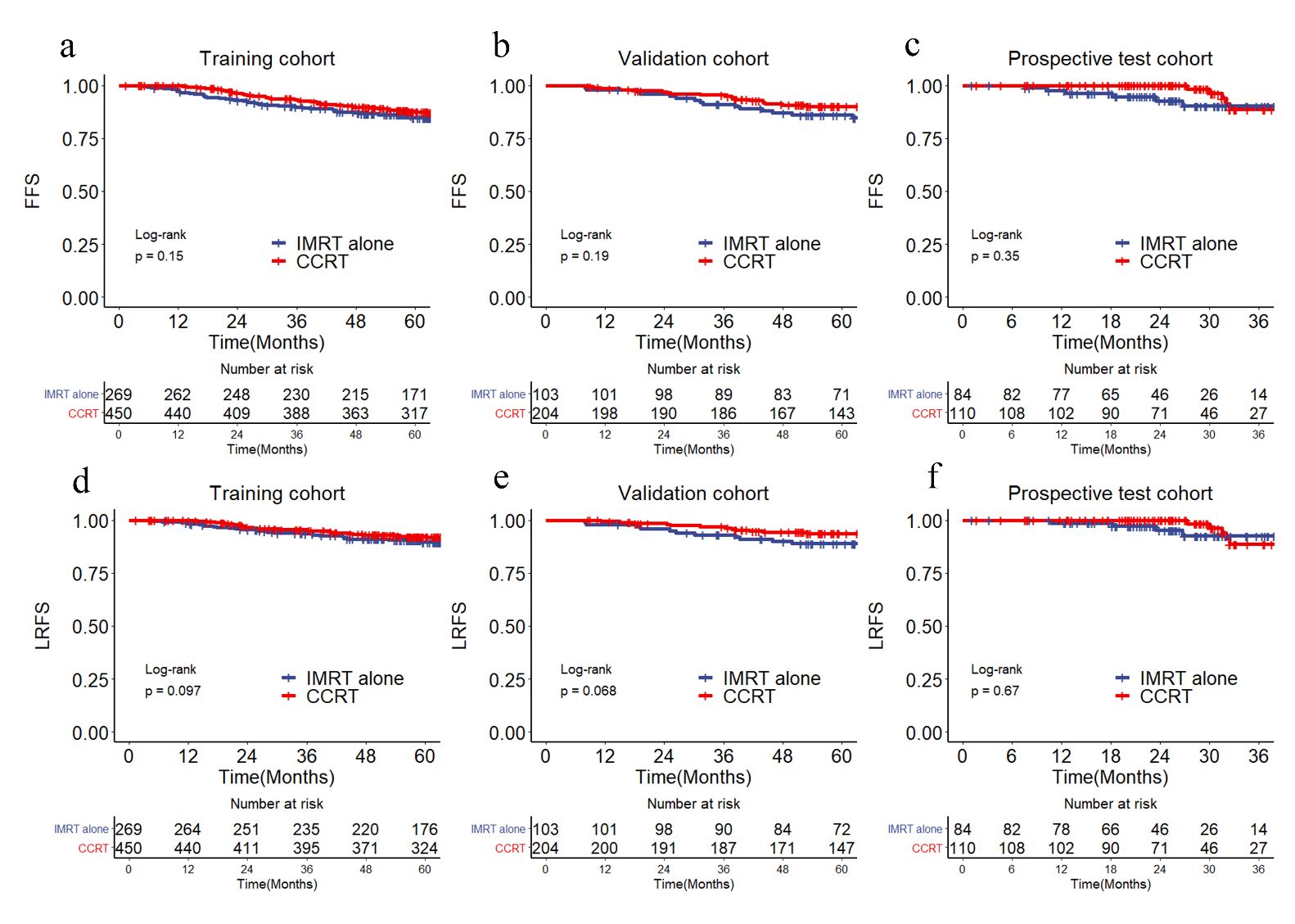


**S5 Fig.** Survival curves of CCRT and IMRT alone for LRFS in the high-risk group (a-c) and low-risk group (d-f) of training, validation, and prospective test cohort

Abbreviations: CCRT, concurrent chemoradiotherapy; IMRT, intensity-modulated radiotherapy; LRFS, locoregional relapse-free survival


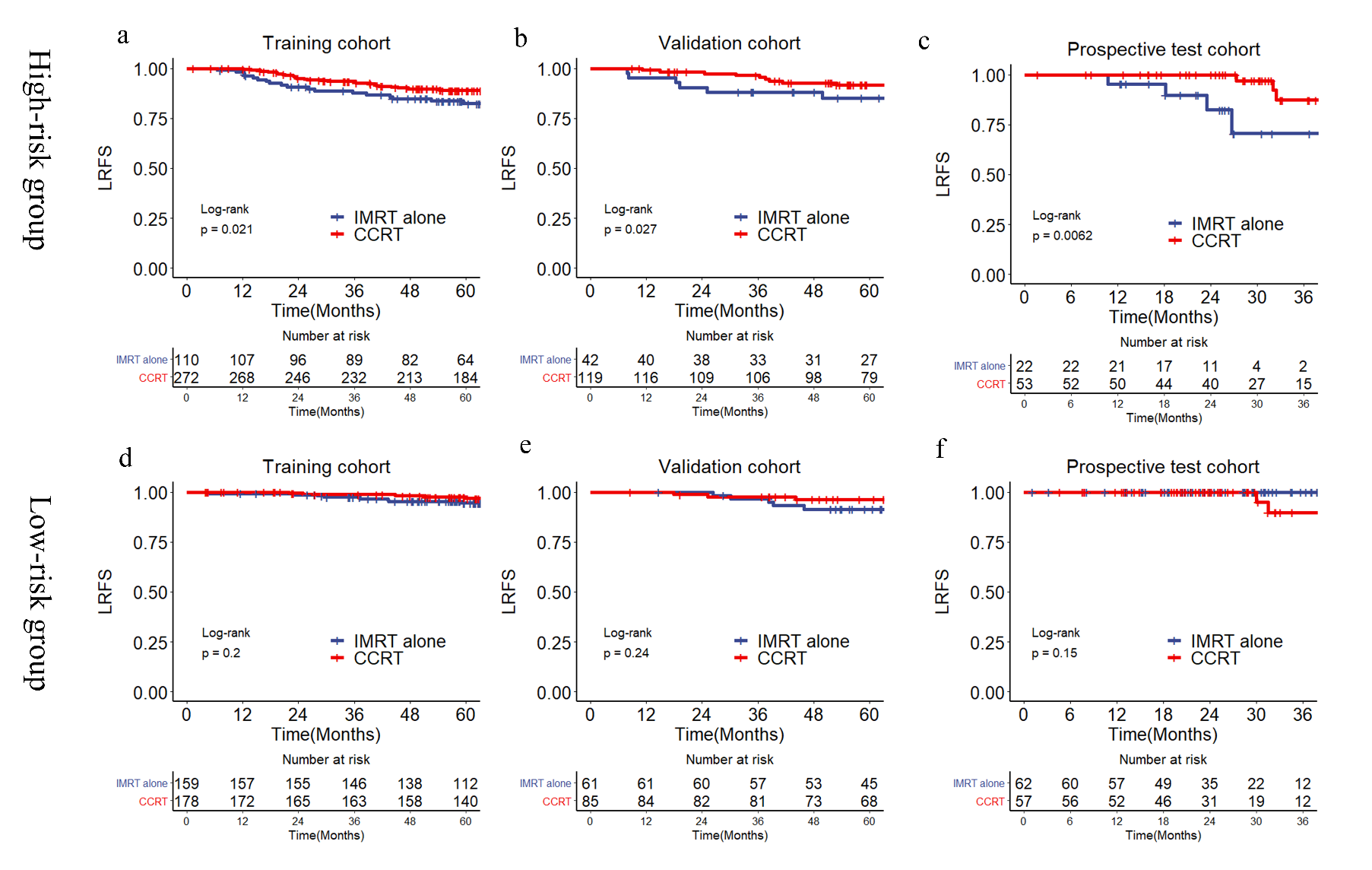


**S6 Fig.** Survival curves of T1N1M0, T2N0M0, and T2N1M0 for (a) DMFS, (b) FFS, (c) LRFS

Abbreviations: DMFS, distant metastasis-free survival; FFS, failure-free survival; LRFS, locoregional relapse-free survival


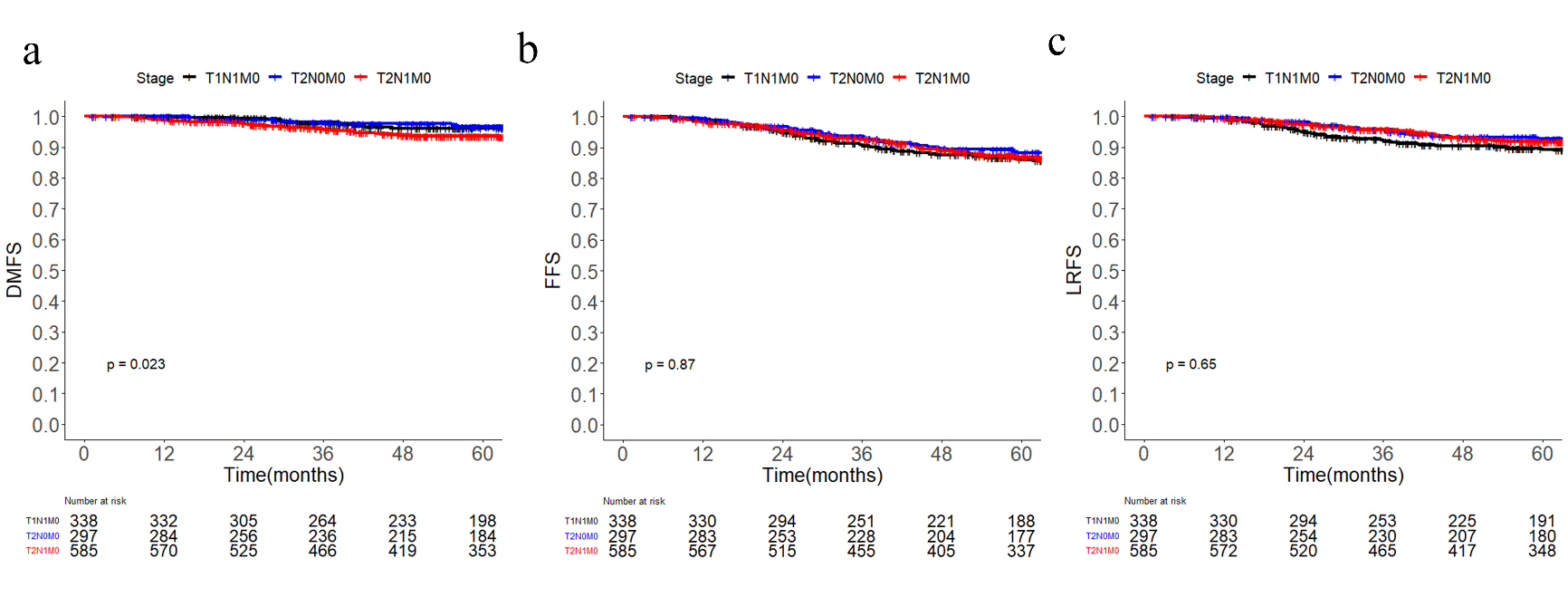


**S7 Fig.** Kaplan–Meier FFS curves of CCRT and IMRT alone for different risk groups. (a) high-risk T1N1M0 subgroup, (b) high-risk T2N0M0 subgroup, (c) high-risk T2N1M0 subgroup, (d) low-risk T1N1M0 subgroup, (e) low-risk T2N0M0 subgroup, (f) low-risk T2N1M0 subgroup

Abbreviations: CCRT, concurrent chemoradiotherapy; FFS, failure-free survival; IMRT, intensity-modulated radiotherapy


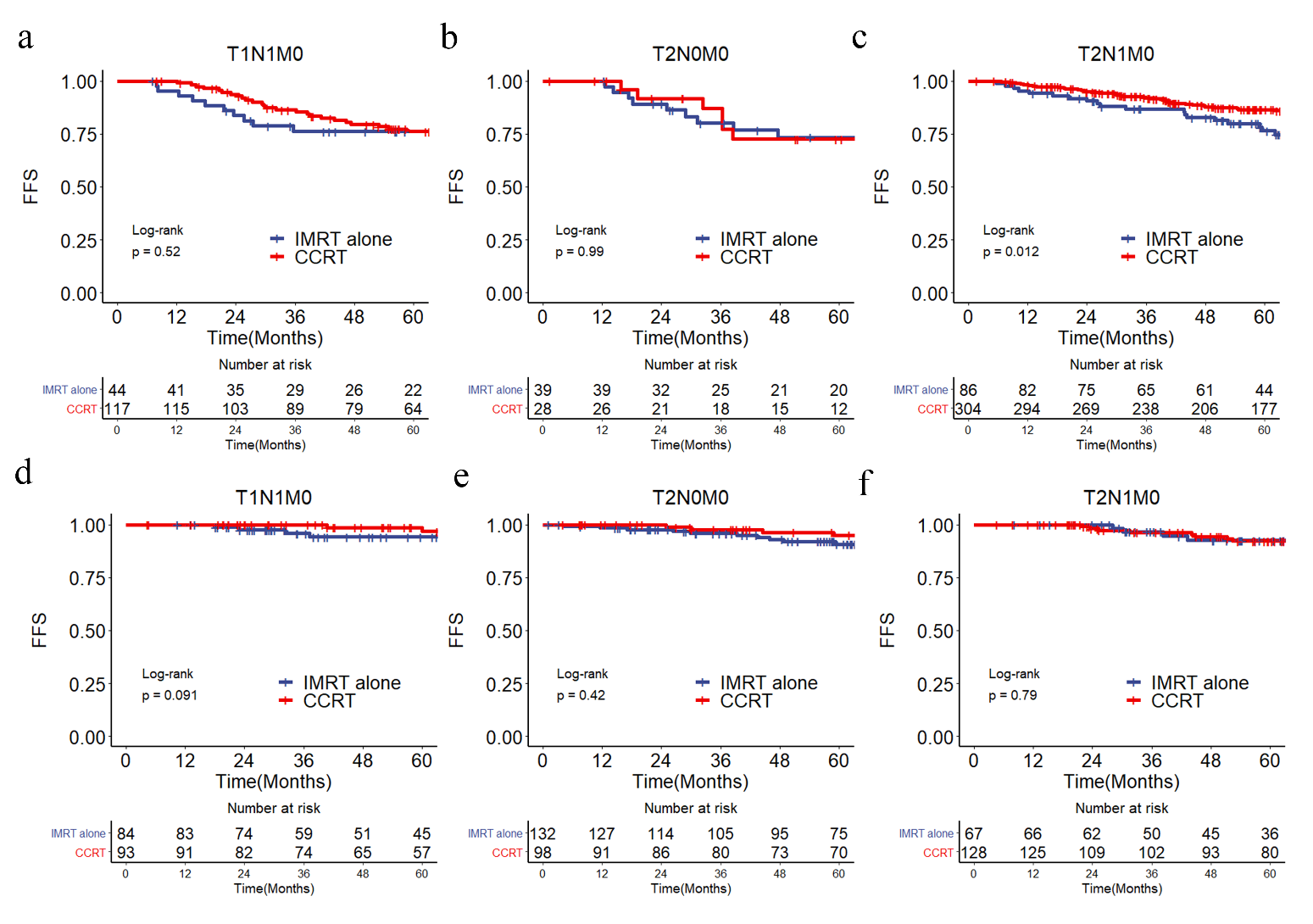


**S1 Table**. Baseline characteristics of T1N1M0, T2N0M0, and T2N1M0 in the whole cohort (N=1220)

|  |  | T1N1M0 | T2N0M0 | T2N1M0 |
| --- | --- | --- | --- | --- |
|  |  | N=338 | N=297 | N=585 |
| Sex |  |  |  |  |
|  | Female | 95 (28.1) | 73 (24.6) | 186 (31.8) |
|  | Male | 243 (71.9) | 224 (75.4) | 399 (68.2) |
| Age |  |  |  |  |
|  | <45 | 188 (55.6) | 114 (38.4) | 288 (49.2) |
|  | ≥45 | 150 (44.4) | 183 (61.6) | 297 (50.8) |
| Pathology |  |  |  |  |
|  | WHO I | 0 (0.0) | 0 (0.0) | 1 (0.2) |
|  | WHO II | 6 (1.8) | 6 (2.0) | 5 (0.9) |
|  | WHO III | 332 (98.2) | 291 (98.0) | 579 (99.0) |
| EBV DNA (copy/mL) |  |  |  |  |
|  | <2000 | 265 (78.4) | 271 (91.2) | 401 (68.5) |
|  | ≥2000 | 73 (21.6) | 26 (8.8) | 184 (31.5) |
| Hemoglobin (g/L) |  |  |  |  |
|  | <120 | 29 (8.6) | 19 (6.4) | 45 (7.7) |
|  | ≥120 | 309 (91.4) | 278 (93.6) | 540 (92.3) |
| LDH(U/L) |  |  |  |  |
|  | <250 | 327 (96.7) | 281 (94.6) | 563 (96.2) |
|  | ≥250 | 11 (3.3) | 16 (5.4) | 22 (3.8) |
| Total tumor volume(mL) |  |  |  |  |
|  | <11 | 207 (61.2) | 260 (87.5) | 249 (42.6) |
|  | ≥11 | 131 (38.8) | 37 (12.5) | 336 (57.4) |
| Treatment |  |  |  |  |
|  | IMRT alone | 128 (37.9) | 171 (57.6) | 153 (26.2) |
|  | CCRT | 210 (62.1) | 126 (42.4) | 432 (73.8) |
| Group |  |  |  |  |
|  | Low-risk group | 177 (52.4) | 230 (77.4) | 195 (33.3) |
|  | High-risk group | 161 (47.6) | 67 (22.6) | 390 (66.7) |

Abbreviations: CCRT, concurrent chemoradiotherapy; EBV, Epstein Barr virus; IMRT, intensity-modulated radiotherapy; LDH, lactate dehydrogenase
